# A Simple Mechanical Method to Modulate the Electrochemical Electrosorption Processes at Metal Surfaces

**DOI:** 10.3390/molecules24203662

**Published:** 2019-10-11

**Authors:** Aiting Yuan, Haixia Zhang, Qibo Deng

**Affiliations:** Institute for New Energy Materials & Low-Carbon Technologies, School of Materials Science and Engineering, Tianjin University of Technology, NO.391 Binshui West Street Xiqing District, Tianjin 300384, China; yuanaiting0613@163.com (A.Y.); haixia0202@126.com (H.Z.)

**Keywords:** mechanical bending, large static strain, metal thin films, electrosorption process, potential-strain response

## Abstract

The coupling of electrochemical processes and surface strain has been widely investigated in the past. The present work briefly introduces a simple method to modulate the electrochemical process at metal surfaces by mechanical bending. In this way, the static strain at the metal layer can reach the order of 1%. The cyclic voltammogram was used to study the electrosorption process of oxygen species at sputtered metal surfaces under different strain states. The experimental results show that the desorption peak potential of oxygen at the Au surface shifted positively by tensile strain, whereas the desorption peak potential at the Pt surface shifted negatively. This phenomenon indicates that tensile strain has an opposite effect on the electrosorption process for Au and Pt surfaces. Our results agree with the previous reports on the potential variation induced by dynamic strain. This work thus offers a simple method to modulate the electrosorption process at metal surfaces and then to enhance the reactivity of metal electrodes.

## 1. Introduction

The electrocatalytic activity of electrode materials is certainly important in the field of energy-related issues. More and more strategies have been developed to extremely enhance the activity, e.g., novel microstructures, single atom materials, and composition effects [1,2,3,4,5,6,7,8]. Recently, the coupling of surface mechanics and the electrochemical process has been considered an interesting topic and offers a new understanding in the field of electrochemistry [9,10,11,12,13,14]. More importantly, surface strain can promote the electrochemical performance of electrode materials [15,16,17,18,19,20,21,22]. Such surface strain can be achieved in experiments by monolayer foreign atom electrodeposition on different support substrates, like the experimental studies in [23,24]. Due to the lattice misfit of crystals, the surface atoms at such a monolayer can be compressive or tensile. In those studies, the foreign monolayer is generally obtained using the underpotential deposition (UPD) method. It is noted that the technique is rather cumbersome and not all metal monolayers can be achieved in experiment by the UPD method. Furthermore, the electrochemical properties are still affected by the electron exchange of the monolayer surface with the underlying substrate by the UPD method. As an alternative method, the surface strain can be achieved by mechanical strategy. Recent studies about the dynamically mechanical strain indicate that the mechanical method can significantly change the electrocatalytic activity of the electrode material of interest [25,26,27]. However, it is inconvenient in practical applications using the dynamic strain induced by the mechanical stretching.

In this work, we introduce a simple mechanical method to modulate the electrochemical process at the metal electrode surface. The metal (Au or Pt) thin films were taken as the examples which were sputtered on the polymer substrates. The electrodes were bent mechanically while heating at 70 °C in water due to the thermal deformation characteristics of polymer substrates. After cooling, the metal layer could keep the strained state. In this way the static strain at the metal layer could reach the order of 1%, which was further determined by X-ray diffractometer (XRD) measurement. The cyclic voltammogram was used to study the electrosorption process of oxygen species at the sputtered metal surface under different strain states. The electrosorption of oxygen species is the focus of this study since it is one of the important steps in the oxygen reduction reaction for the fuel cell. 

## 2. Results and Discussion

**Figure 1:**
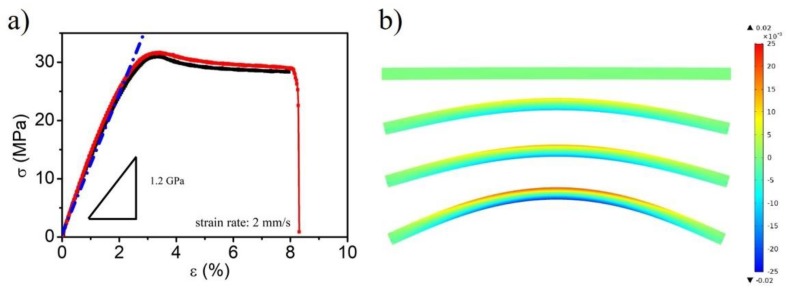
The mechanical properties of the polylactic acid (PLA) substrate (**a**) and the strain simulation map of the electrode under different mechanical bending (**b**).

The mechanical properties of the polymer (polylactic acid, PLA) substrate was examined. The results are shown in Figure 1a, and the value of the Young modulus yielded 1.2 GPa. Using finite element analysis, Figure 1b exhibits the strain distribution map of the sample under different mechanical bending. It indicates that the surface strain amplitude was controllable under mechanical bending. 

**Figure 2:**
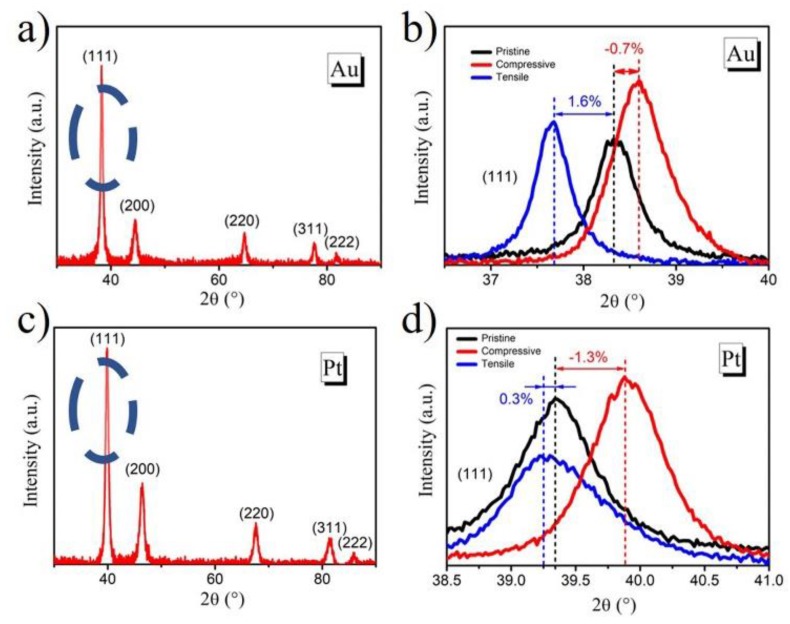
The X-ray diffractometer (XRD) patterns of the as-sputtered Au (**a**) and Pt (**c**) film electrodes. The (111) crystal dominant planes of Au (**b**) and Pt (**d**) electrodes under different strain states.

The X-ray diffractometer (XRD) was used to detect the crystal orientations of the metal thin films (Au and Pt) under different strain states, as shown in Figure 2. The diffraction peaks corresponded to the (111), (200), (220), (311), and (222) crystal planes of the Au and Pt film electrodes, respectively. It was clearly shown that the (111) crystal plane was dominant in this study (see Figure 2a and c). Thus, we paid attention to the shift of diffration angle (2θ) for the (111) plane under the different strain states, which were induced by mechanical bending. From the results of Figure 2b (Au) and Figure 2d (Pt), the angle of the (111) crystal plane positively shifted under the compressive strain whereas the 2θ negatively shifted under the tensile strain. 

According to Bragg’s equation, the diffration angle can be used to calculate the d-spacing value:

(1) 2dsinθ=nλ.

It is seen that the crystal lattice can be changed by the compressive or tensile strain. There is a smaller d-spacing in the compressively strained lattice and a larger d-spacing value of the tensilely strained Au (or Pt) lattice [28,29].

d_0_ is the (111) d-spacing of the pristine Au (or Pt) and d is the (111) d-spacing of the strained Au (or Pt). Therefore, surface strain, ε, can be calculated from XRD data:

(2) $$\varepsilon =\frac{d-d_{0}}{d_{0}}=\frac{\sin\theta_{0}-\sin\theta }{\sin\theta }.$$

As examples, the tensile strain of the Au film electrode reached 1.6% and the compressive strain reached −0.7%, as shown in Figure 2b, according to Equation (2). The tensile strain of the Pt film electrode reached 0.3% and the compressive strain reached −1.3%, as seen in Figure 2d. 

**Figure 3:**
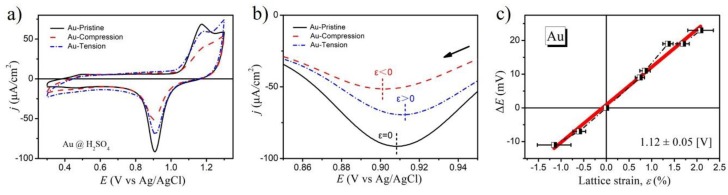
(**a**) Cyclic voltammogram (CV) curves of the Au surface under different strain states at 10 mV·s^−1^ in 10 mM H_2_SO_4_; (**b**) Oxygen desorption peak of the Au electrodes; (**c**) The desorption potential variation is plotted as a function of the lattice strain.

The electrochemical performance of Au films was measured in 10 mM H_2_SO_4_ aqueous solution by conventional cyclic voltammogram (CV). The measurements were performed at a potential range between 0.3 V and 1.3 V (vs. commercial Ag/AgCl) with a scan rate of 10 mV·s^−1^ at room temperature. The potential range was chosen to cover the electrosorption process of oxygen species. As seen in Figure 3a, the shapes of the CV for different strain states of Au thin film were nearly identical, which indicated that the mechanical bending strain did not affect the crystal structure. 

The desorption potential peak of oxygen species was shifted by different lattice strains. The enlarged figure in Figure 3b shows the peak potential value of oxygen desorption shifted to the negative potential under compressive strain, while it shifted to the positive potential under tensile strain. When plotting the change of the peak potential for oxygen desorption (relative to the pristine state) as a function of lattice strain, which was calculated from the XRD data, there was almost a linear relationship within the error bar (see Figure 3c). The best linear fitting obtained a slope value of 1.12 ± 0.05 V. The positive sign of electrocapillary coupling coefficient agreed with the cantilever bending experiment [13]. Our work directly quantified the electrocapillary coupling coefficient of the Au surface at the oxygen desorption potential.

**Figure 4:**
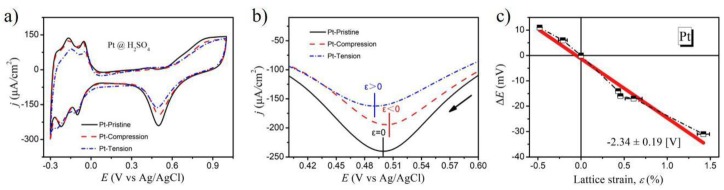
(**a**) CV curves of the Pt surface under different strain states at 10 mV·s^−1^ in 10 mM H_2_SO_4_; (**b**) Oxygen desorption peak of the Pt electrodes; (**c**) The desorption potential variation is plotted as a function of the lattice strain.

Since the electrocapillary coupling coefficient for Pt metal near the electrosorption process of oxygen species has been well investigated, the Pt electrode was also examined in a similar way to the Au metal. The CV curves were performed in 10 mM H_2_SO_4_ at a potential scan rate 10 mV·s^−1^ between −0.3 V and 1.0 V (vs. Ag/AgCl) at room temperature, and the results are shown in Figure 4a. As seen in Figure 4b, the desorption peak potential of oxygen species shifted to the negative potential under tensile strain, whereas that potential value positively shifted under compressive strain. Figure 4c shows the linear relationship between the change in desorption potential and lattice strain. The best linear fit yielded slope values of −2.34 ± 0.19 V. The negative value of the coupling parameter, potential-strain response near the oxygen desorption, was in good agreement with published reports [30].

**Figure 5:**
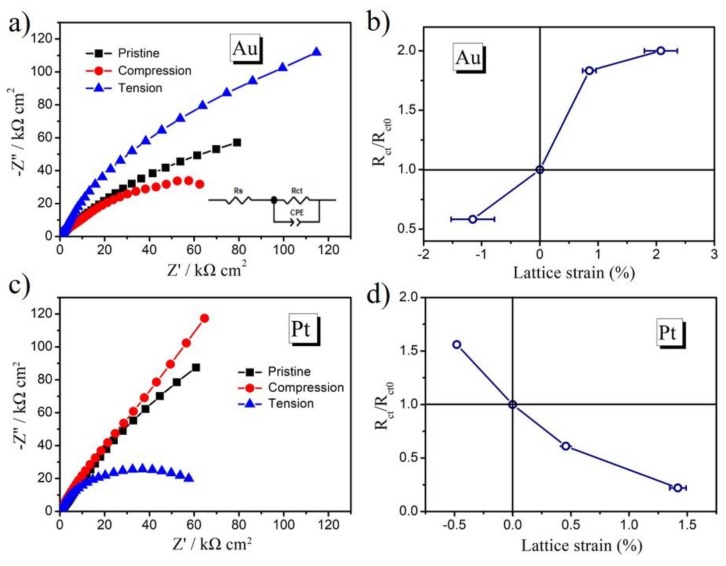
(**a)** Electrochemical impedance spectroscopy (EIS) spectra of the Au film; (**b**) The relationship of lattice strain and the R_ct_ variation of Au film; (**c**) EIS spectra of the Pt film; (**d**) The relationship of lattice strain and the R_ct_ variation of Pt film.

In order to further understand the different effects of surface strain on the Au and Pt electrode surfaces, electrochemical impedance spectroscopy (EIS) was investigated. The EIS measurements were chosen at the electrode potential of 950 mV (vs. Ag/AgCl) for Au electrode and of 600 mV for Pt electrode with a sweep frequency from 0.01 Hz to 100 kHz at room temperature. The EIS data is shown in Figure 5. The EIS diagram is equipped with appropriate equivalent circuits to further understand the effect of mechanical strain on the peak potential, as shown in the inset of Figure 5a [31,32]. The resistor Rs in the simple equivalent circuit is attributed to the solution impedance. R_ct_ is attributed to the charge-transfer resistance in the Au and Pt catalysts.

The R_ct_ value of Au film under the compressive strain was smaller than the pristine Au film, while the tensile strain had a larger R_ct_. This indicated that tensile strain was more efficient for charge transfer in the Au film catalyst. Figure 5b plots the ratio of R_ct_ variation as a function of lattice strain, showing a positive correlation. For the case of the Pt electrode, R_ct_ from the EIS spectra (Figure 5c) decreased with application of tensile strain at the electrode surface. This indicated that compressive strain was more efficient for charge transfer in the Pt film catalyst. Figure 5d shows the negative correlation when the ratio of R_ct_ was plotted as a function of lattice strain.This trend agreed with the electrochemical impedance data of Au and Pt surfaces in previous reports [33,34]. The EIS data support the findings of strain-shifted desorption potential for Au and Pt metals in this study.

## 3. Materials and Methods 

The substrate materials (polylactic acid, PLA) were cut into the size of 1 × 2 × 0.07 cm and cleaned by ultrasonic for several times in ethanol (99.99%, Tianjin Hengshan Chemical Technology Co., Ltd, Tianjin, China) and ultrapure water (18.2 MΩ cm^−1^), respectively. After drying in vacuum oven at room temperature for 20 h, different coatings were deposited on the polymer substrates using the magnetron sputtering apparatus (SKY Technology Development Co., Ltd., Shenyang, China). 

In this study, Ti film and Au or Pt film were deposited on the polymer substrate, and the preparation process in detail was as follows: Ti film as a wetting layer was firstly deposited on the polymer substrate using a Ti target (99.99%, Beijing Zhongnuo New Material Technology Co., Ltd, Beijing, China) by direct current (DC) magnetron sputtering. The Ti sputtering was performed with sputtering power of 20 W and sputtering time of 2 min. The thickness of the Ti film was about 10 nm. Then the Au film was sputtered on the Ti coated substrate using an Au target (99.99%, Beijing Zhongnuo New Material Technology Co., Ltd, Beijing, China) by radio-frequency magnetron sputtering. The sputtering power was 20 W and sputtering time was 4 min. The thickness of gold film was about 30 nm. Similarly, the Pt film was also sputtered on the Ti coated substrate using Pt target (99.99%, Beijing Zhongnuo New Material Technology Co., Ltd, Beijing, China) by radio-frequency magnetron sputtering with the sputtering power of 20 W and sputtering time of 5 min. The thickness of Pt film was about 30 nm. Before the sputtering, the vacuum degree of the sputtering chamber was 5 × 10^−4^ Pa. The working pressure was 2.0 Pa and the Ar gas (99.99% purity) flowmeter revealed 50 sccm. All the targets were pre-sputtered for ten minutes to wipe off the impurities on the surface of the targets. 

**Figure 6:**
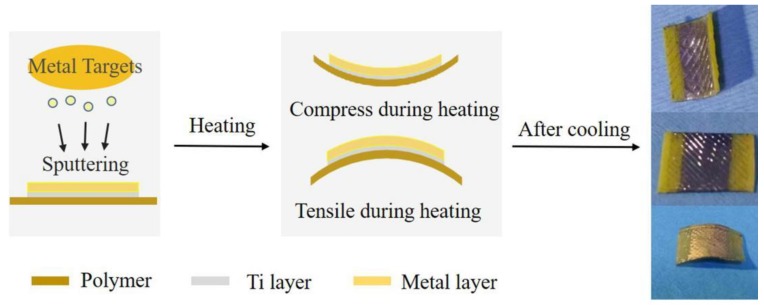
Schematic diagram showing the preparation of strained metal.

In order to measure the effect of different strain states on the electrochemical electrosorption processes, the electrodes needed to undergo heat treatment. The samples were heated in ultrapure water at 70 °C for 5 min. During heating, the working electrode was clamped between a fixed and a mobile grip, the latter being displaced by mechanical bending. After cooling in the air at room temperature for 5 h, the electrode surface generated different strain states by the different bending forces. The schematic diagram is shown in Figure 6. The lattice strain states were characterized by X-ray diffractometer (XRD, Rigaku D/max-2500, Rigaku Corporation, Shoshima, Tokyo, Japan) with Cu Kα radiation, collecting data with a scanning speed of 8 °/min and a step size of 0.02° in an angular range 2θ of (10° < 2θ < 90°). Selecting three positions of the pristine and deforming metal film electrode was characterized, and the average diffraction angle calculated. Considering that the maximum elastic strain of substrate was about 2% (Figure 1a), the maximum surface strain in the present study was 2% for the Au electrode and 1.5% for the Pt electrode due to different Young’s moduli of the metal layers.

In our experiment, electrochemical data were obtained from an electrochemical workstation (CHI760E, Shanghai Chenhua Instrument Co., Ltd, Shanghai, China). The electrochemical measurements were conducted at room temperature using a typical three-electrode system that used the sputtered metal film as a working electrode, a carbon rod as a counter electrode, and a commercial Ag/AgCl electrode as the reference electrode. The aqueous solution of 0.01 M H_2_SO_4_ was used as the electrolyte. Cyclic voltammetry (CV) was performed at room temperature at scan rates of 10 mV·s^−1^. The electrolyte was deaerated with high-purity (99.99%) N_2_ for approximately 30 min before CV measurement. Electrochemical impedance spectra (EIS CHI760E, Shanghai Chenhua Instrument Co., Ltd, Shanghai, China ) was obtained in a frequency range from 0.1 Hz to 100 kHz with an AC amplitude of 5 mV.

## 4. Conclusions

In brief, a simple mechanical method was explored to modulate the electrochemical process of metal surfaces. The static strain could reach the order of 1%, which was further confirmed by the XRD technique. The electrochemical impedance data support the finding of opposite effects of strain on Au and Pt metals for the electrosorption process of oxygen species. Since the method of applying strain on the metal surface is quite simple, it is convenient in practical applications for enhancing the electrocatalytic activity of metal electrodes.
